# Structural Response of High-Strength Wire-Reinforced UHPC Slabs Subjected to Bending

**DOI:** 10.3390/ma15217550

**Published:** 2022-10-27

**Authors:** Wangcheng Luo, Xiaoyong Luo, Jun Wei, Dinghao Sun

**Affiliations:** 1 School of Civil Engineering, Central South University, Changsha 410075, China; 2 Prefabricated Construction Engineering and Technological Research Center of Hunan Province, Changsha 410075, China

**Keywords:** ultra-high performance concrete (UHPC), structural performance, high strength steel wire, theoretical method, parametric analysis

## Abstract

Using high strength wire (HSW) as a longitudinal reinforcement in UHPC can make full use of the outstanding properties of UHPC. In this paper, the flexural test was carried out on normal rebar-reinforced UHPC (NRRU) and HSW reinforced UHPC (HSWRU) slabs. The cracking resistance, failure modes, bearing capacity and deformation characteristics of specimens were investigated. The test results indicated that both HSWRU and NRRU specimens exhibited excellent flexural performance under concentrated loads. Fewer inclined cracks and a slower cracking development process were observed for HSWRU specimens, and brittle failure did not occur during the whole loading process. As compared to HSWRU specimens, the cracking and ultimate load of NRRU specimens increased by 24.64% and 85.47%, respectively, due to a higher reinforcement ratio. Then the theoretical method available for flexural capacity and ductility calculation was proposed, and the feasibility was substantiated through test results. In addition, the traditional deformation ductility coefficient was found to be 30% conservative against the applied energy ductility coefficient. Finally, the extensive parametric analysis revealed that the increase of the reinforcement ratio and the strength of the steel rebar significantly enhanced the ultimate capacity, while the ductility coefficient was obviously weakened. Inversely, those two factors had little impact on the cracking capacity. Moreover, section height was found to be beneficial for both the flexural capacity and ductility of specimens.

## 1. Introduction

Ultra-high performance concrete (UHPC) is a new generation of cement matrix composite material based on the densely packing theory. Owing to the constraints of coarse aggregate size and the addition of steel fiber, the reduced inner prosperity and local fracture zone are formed in the mixture [1]. Typically, UHPC offers a compressive strength of over 150 MPa and tensile strength of over 5 MPa, higher post-crack tensile strength [2] and improved post-cracking behavior [3] without brittleness grow due to the “strain-hardening” phenomenon in fiber-reinforced UHPC [4], and the superiority of UHPC structures over normal strength concrete (NSC) has already been documented in previous studies [5,6]. It should be noted that recent studies on UHPC include reactive powder concrete (RPC), ultra-high performance fiber reinforced concrete (UPFRC) and high ductility cement-based composites (HDCC) [7]. Given the advanced mechanical properties, UHPC has been increasingly employed in civil engineering to improve the structural responses of structures.

Structure steel with a nominal yield strength higher than 500 MPa is denoted as high strength steel (HSS) [8]. In recent years, higher strength structural steel is urgently needed for engineering applications to satisfy the frequently growing requirements of building and bridge constructions. Components using HSS instead of normal strength steel (NSS) can provide structures with higher strength, smaller size and reduced dead load; thus, the quality of produced components is enhanced and the construction speed is accelerated, especially to meet the demands of a widely popularized assembly construction [9]. Moreover, HSS was identified as having the potential to enhance the mechanical behavior of structures, since new appealing features were found through conducted investigations [10]. Ban et al. [11,12,13] experimentally tested HSS-restrained columns under axial load. The results showed that the overall buckling performance was significantly improved over normal strength steel components. In addition, Chiew et al. [14] experimentally tested the fatigue performance of two full-scale HSS built-up box joints. Giro et al. [15] studied the endplate-connecting performance of HSS, the nonlinear behavior and ductility analysis and the validation of current specifications were included in this research. Considering their mutually owed superior mechanical properties, using HSS or high strength wire (HSW) as a reinforcement in UHPC to fully exploit its superior properties has today gained much attraction from researchers [16]. However, few findings are available on the structural responses of HSS- or HSW-reinforced UHPC members in detail.

Over the past decades, many researchers have expounded on the structural behaviors of reinforced UHPC (RUHPC) to better explore its advanced mechanical properties so that the design method can be optimized. Meanwhile, Yin et al. [17] have conducted flexural tests on hybrid steel-UHPC slabs with different UHPC thickness. ZHANG et al. [18] evaluated the flexural performance of steel-UHPC composite beams with stud connections at the interface, the bearing capacity and the deformation characteristics of steel-UHPC composite beams with stud connectors (SU-S) and bolt connectors (SU-B) were investigated in detail. Qi et al. [19] tested two large-scale steel-ultra high performance fiber-reinforced concrete (UHPFRC) composite beams and one normal concrete beam. Results indicated that both the stiffness and crack control ability was improved via UHPFRC. On the other hand, to provide further design guidance, the theoretical and numerical analysis of RUHPC structures should be addressed. Shirai et al. [20] proposed a novel way to predict the flexural strength of UHPC strengthened composite structures, and the parametric study verified the applicability of proposed method. Moreover, the existing short-term stiffness calculation method of RUHPC was revised by Xu et al. [21,22]. Dogu et al. [23] and Zhang et al. [24] constructed the analytical equations for the flexural calculation of both post-tensioned RUHPC beams and UHPC-RC slabs. To be noted, finite element models were established to verify the generated analytical methods [25,26]. Those studies demonstrated that RUHPC members exhibited excellent structural performance when subjected to external loads, and corresponding analytical models were proposed through the collected experimental information.

Recently, investigations have been carried out by researchers to promote the application of high strength rebar reinforced UHPC (HSRU) members. Samaneh et al. [27] studied the bond behavior of HSRU with different bond length. It was reported that the HSS provided a higher bond tension to concrete, and UHPC can significantly reduce the embedded length of rebars. The shear behavior of connectors in HSRU composite beams with different diameters and configurations was experimentally investigated by Tong et al. [28]. Results showed that the shear capacity and shear stiffness of UHPC components were significantly enhanced compared with NSC, and formulas for shear stiffness calculation were also proposed. Saleem et al. [29] developed a new type of slight bridge deck reinforced with HSS. The experimental results showed that the new system exhibited superior mechanical properties, and most specimens failed in shear but not as suddenly as common shear failure. Xia et al. [30] analyzed the shear failure model of HSRU beams, and the calculation method of the shear capacity was proposed. In addition, Ghasemi et al. [31] optimized the existing UHPC-HSS bridge deck system and the tested specimens represented improved mechanical properties.

In general, benefits in terms of both structural and mechanical performance can be foreseen when adopting HSRU in structures to make full use of its excellent mechanical properties. Aforementioned issues implies that the majority of existing research focused on the structural performance of normal rebar reinforced UHPC (NRRU) members and mechanical aspects of HSRU and some merely concerned the structural behavior of single steel [32,33,34]. Nevertheless, the structural behavior and theoretical analysis of high strength wire reinforced UHPC (HSWRU) slabs remain very limited. Moreover, a comprehensive review conducted by Sharifa et al. [35] revealed that the RUHPC slabs exhibited excellent structural performance and thereby had broad application prospect in the future. Therefore, more investigations are required to assess the potential advantages of HSWRU slabs in broader construction fields.

In response, the experimental test of the newly developed HSWRU and NRRU slab specimens were performed in this paper. In this case, structural behavior was further investigated under the two loads concentrated in the third of span, then the theoretical models for flexural capacity and ductility coefficient calculation were proposed. Finally, the comprehensive parametric analysis was performed to uncover the effect of each parameter on the structural responses of specimens.

Through the conducted investigation, the test results were described in detail and theoretical analysis was clearly expounded: (1) crack development process and failure discover; (2) curves of bending load versus mid-span deflection of tested slabs; (3) curves of bending load versus mid-span strain of tested slabs; and (4) theoretical calculation method for the evaluation of structural response and validation; as well as (5) parametric analysis on effect factors of structural response. Finally, the influences of the reinforcement ratio, strength of steel rebar and section height on flexural capacity and ductility index action were ascertained.

## 2. Experimental Program

### 2.1. Design and Description of Specimens

**Figure 1:**
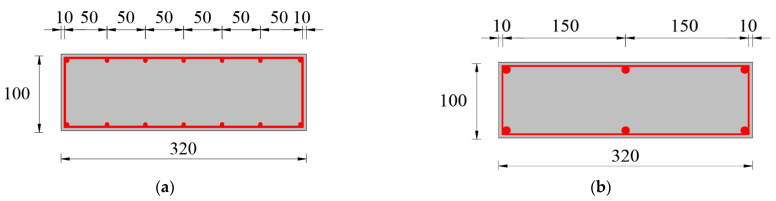

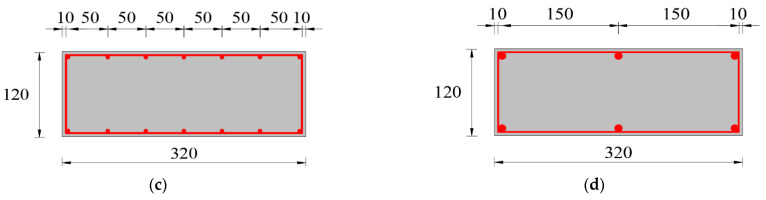
Design of test specimens: (**a**) B1, (**b**) B2, (**c**) B3, (**d**) B4 (unit: mm).

A total of four test slabs were fabricated and tested under the four-point bending load, consisting of NRRU slab specimens labelled B2 and B4, respectively, as the standard group. The contrast group comprised two HSWRU slab specimens labelled B1 and B3, respectively. The standard group was designed for basic structural behavior observation, while the contrast group was aimed to uncover the use of HSW on the structural response and coordination effect of RUHPC specimens. The primary dimensions of each slab were 650 mm in total length (*l*) and 320 mm in width (*b*), and the reduced size was designed to better explore the superior properties of UHPC. B1 and B2 were deliberately cast at 100 mm in section height (*h*), and B3 and B4 were 120 mm to vary in the volume of steel fiber. The geometric sizes and layout of reinforcement is illustrated in Figure 1. The center of the support was 25 mm away from both sides of the specimen and the two loading points of the distribution beam acted on a third of the span, therefore a 200 mm pure-bending span was formed between the loading position, and the 600 mm calculating span (*l_0_*) was also formed with 25 mm cantilever segments on both sides.

The reinforcement ratio (*ρ*) should be higher than NSC specimens when adopting normal strength steel to fully exploit the outstanding properties of UHPC and form the constraint skeleton of steel fiber. A total of 6 Φ20 (Φ is diameter) deformed rebars with a design yield strength of 400 MPa (HRB400) and 14 Φ5 HSW with a designed yield strength of 1270 MPa were thereby adopted as longitudinal reinforcement in NRRU and HSWRU specimens, respectively. The reinforcement ratio were 0.86% and 0.72% for B1 and B3 and 5.89% and 4.91% for B2 and B4, respectively; intervals between the longitudinal rebars were 50 mm for specimens in standard group, while it was 150 mm in contrast group. No transverse reinforcement was used in the test specimens. To avoid the anchorage failure and unwished movement in positioned steel rebars, the stirrups with the 335 MPa design yield strength were used in the reinforcement restraining with the 10 mm thick of UHPC cover. Geometric details of reinforcement are summarized in Table 1.

### 2.2. Material Properties

Table 2 listed the composite matrix of the UHPC used in this study. The binder consisted of commonly used ordinary Portland Cement and silica fume; fly ash was employed as fine aggregates; and quartz powder, slag powder and quartz sand were also used in the mixture. Furthermore, the excellent workability provided by the low water-to-cement ratio was secured by the addition of high-efficiency water-reducing agent, and the concrete was mixed with steel fibers at a volumetric ratio of 2%. The added straight steel fiber had a value of 10 mm in length and 0.2 mm in diameter, whereas the tensile strength was larger than 2000 MPa with the elastic modulus of 210GPa. The properties of steel fiber are illustrated in Table 3. The test results of various steels are shown in Table 4.

### 2.3. Property Tests of Concrete

**Figure 2:**
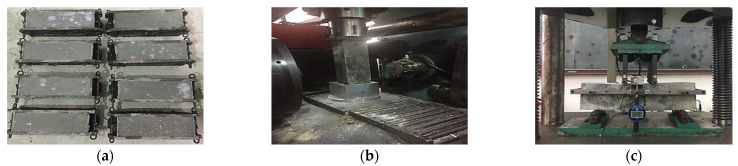
Material tests: (**a**) specimen preparation, (**b**) compressive test, (**c**) flexural–tensile test.

According to the national standards [36], three prisms in size of 100 mm × 100 mm × 300 mm were poured to measure the compressive strength (*f*_c_) under axial compressive tests, and the same specimens were adopted for elastic modulus (*E*) tests. Moreover, the flexural–tensile tests were conducted for three prisms in size of 100 mm × 100 mm × 400 mm, and the flexural (*f_fl_*) and tensile (*f_t_*) strengths were obtained by using Equations (1) and (2), according to [37], where *F*_n_ is the value of applied load and *a* is a third of the clear span, whose value is equal to 100 mm in this test. Figure 2 clearly shows the procedure of material property tests. The compressive strengths obtained at 28 days for each specimen are collected in Table 5, where *S* is the standard deviation of test specimens.

(1) $$f_{fl}=\frac{3F_{n}}{a^{2}}$$

(2) $$f_{t}=f_{fl}\frac{0.08a^{0.7}}{1+0.08a^{0.7}}\;$$

### 2.4. Experimental Set-Up and Methodology

**Figure 3:**
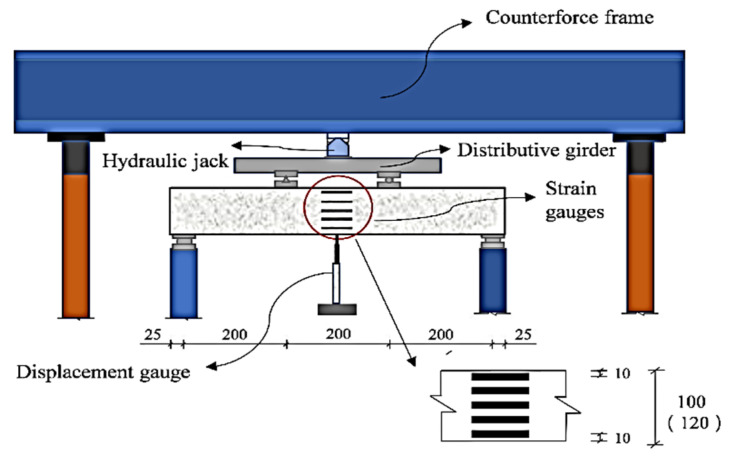
Configuration of monitoring measurements (unit: mm).

Figure 3 shows the configuration of monitoring arrangement. For each test specimen, five longitudinally oriented electrical resistance strain gauges (SG) were symmetrically pasted along the height to observe the changes in the tensile strain, compressive strain and neutral axis position. In this case, two SG were attached onto the corresponding height of longitudinal reinforcement, and the other three were also attached along the surface. Vertical deflection was captured using a linear variable differential transducer (LVDT) with an accuracy of 0.01 mm, which was fixed on a rectangular steel plate and installed beneath the mid-span of specimen. Moreover, crack width was measured by the MG10085-1 reading microscope, and the crack shape was portrayed using the manual marker.

**Figure 4:**
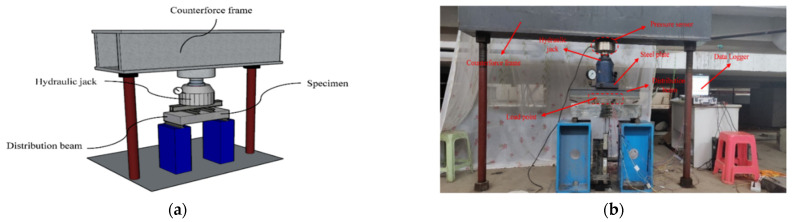
Test setup: (**a**) Schematic diagram of loading device, (**b**) actual loading device.

**Figure 5:**
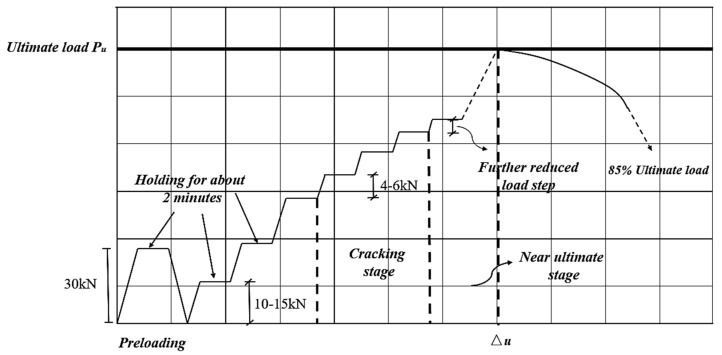
Loading path.

The schematic diagram of the test setup and practical loading device are shown in Figure 4. Each specimen was simply supported at both ends and subjected to the concentrated loads generated by the hydraulic jack through the distribution beam at a loading rate of 0.1 kN/s. Pressure sensors fixed on the jack was instrumented to measure the value of axial load with an accuracy of 0.01 kN. A preload with a load value of 30 kN (Smaller than 40% of the estimated cracking load) was applied prior to the practical test to ensure the integrity and reliability of LVDT, strain gauges and recorders. Furthermore, this procedure was also aimed to verify the uniformity of load transferring from the steel beams to the specimen surface. The load increment before cracking was 10~15 kN and held for approximately two minutes at each load step. When specimens entered the cracking stage, the load step was adjusted to 15%~20% of estimated ultimate load (*P*_u_) until the vertical load dropped to around 85% of *P*_u_. All the readings of the overall process were automatically recorded by the JM3841 static strain test system with a continuous frequency of 1 Hz. The loading path of this test is plotted in Figure 5.

## 3. Experimental Results and Discussion

### 3.1. Crack and Failure Modes

**Figure 6:**
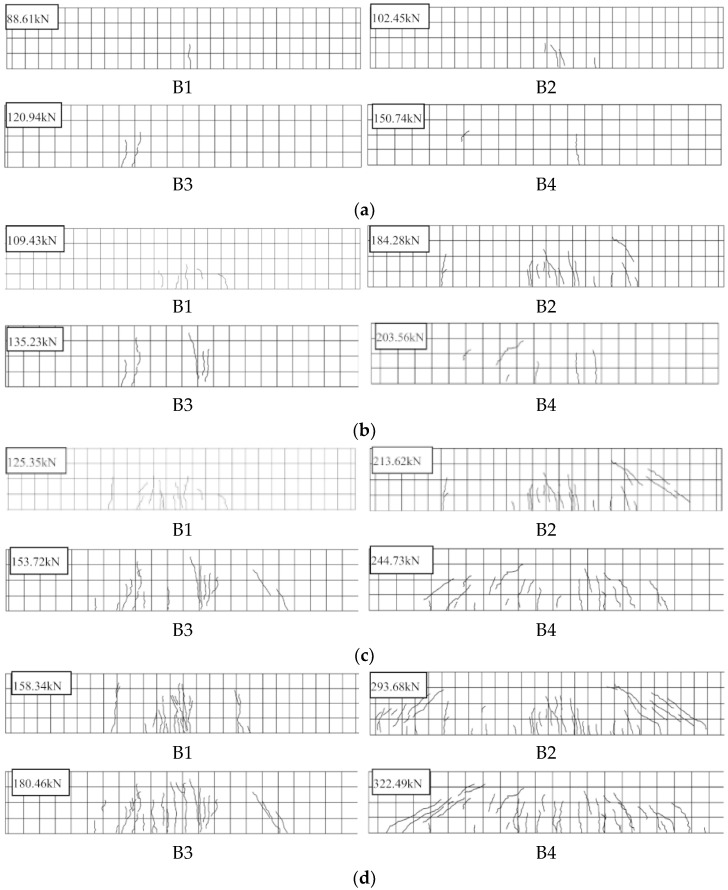
Crack development process of specimens: (**a**) Initial crack, (**b**) crack width 0.1 mm, (**c**) crack width: 0.2 mm, (**d**) failure stage.

Varying in width, the cracking development periods are summarized as shown in Figure 6. It can be inferred that the main cracking process of test slabs included follow periods: (1) During the period of increased linear load, no cracks were observed. Once the load reached around 60% *P*_u_ for HSWRU and 30% *P*_u_ for NRRU, respectively, the first crack occurred in the pure bending span for all test slabs, and then one extra crack appeared in B3 and B4 as the load gently rose. It is worth mentioning that four basically vertically tended cracks appeared at the bottom of B2 before the width became larger. (2) As the load continuously increased, cracks in the NRRU began to develop slightly beyond the pure bending span and the tendency to extend upward along the height initially emerged. For HSWRU specimens, an increment in both number and width of cracks remained hidden, and cracks were mainly concentrated near the bottom of the specimen, as shown in Figure 6a. When the width measured by the visualizer reached 0.1 mm, the load was about 75% *P*_u_ for HSWRU and 65% *P*_u_ for NRRU, respectively. Simultaneously, other than B2, there were few cracks close to the load point, either the bending span or both sides, as shown in Figure 6b. (3) The load occupied approximately 85% *P*_u_ in HSWRU and 75% *P*_u_ in NRRU specimens when the maximum crack width reached 0.2 mm. As can clearly be seen from Figure 6c,d, both the number and length of cracks increased significantly during this period, and new cracks in HSWRU primarily appeared on the bending span. Except for two inclined cracks near the right-side load point of B3, no obvious inclined cracks occurred in HSWRU. Nevertheless, for NRRU specimens, a few fresh vertical cracks were observed in the middle span, but the inclined cracks developed abruptly at both ends of the specimens as compared to the previous periods, which developed from two sides to the middle span along the section height of specimens. Moreover, the crack width gradually increased in tested specimens and the main crack was not observed during the whole process, while for NRC members, the newly-emerged cracks usually owned a certain width instead, and the main crack was formed when specimen failed.

**Figure 7:**
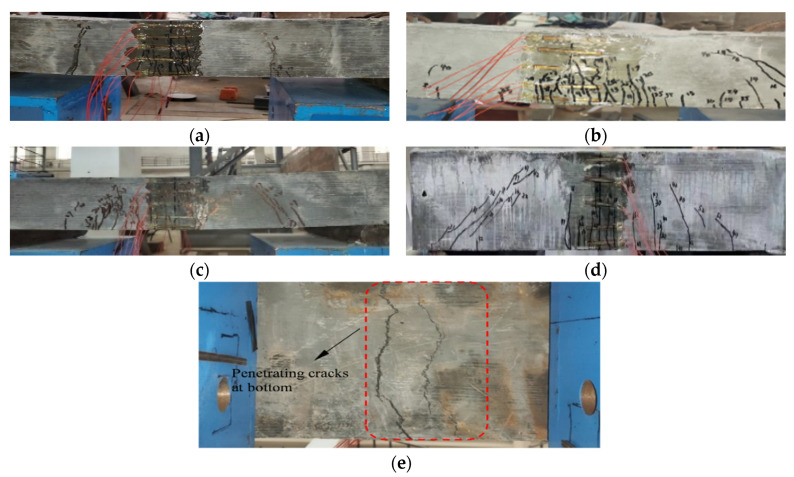
Failure modes of test slabs: (**a**) B1 failure, (**b**) B2 failure, (**c**) B3 failure, (**d**) B4 failure, (**e**) penetrating cracks at specimen bottom.

Figure 7 illustrates the failure mode of each specimen. The specimens at the failure stage were presented in such a manner that vertical cracks were densely distributed near the mid-span section, together with the steel fiber pulled out. As depicted in Figure 7e, when the failure stage was reached, penetrating cracks were formed in the tensile zone at the bottom of the specimens, whose widths were larger than the observed interface cracks. To summarize, the failure process presented as similar among all specimens. Firstly, elastic work was maintained before cracking, then once the load moved beyond the cracking limitation, specimens entered the cracking stage and both vertical and inclined cracks developed rapidly in both length and width and tended to develop in the height direction. When the control load reached the ultimate load, the concrete was partly crushed and the longitudinal bars at the bottom yielded and the specimens failed. Accordingly, the whole failure process of all the test slabs was signified by the plastic behavior.

### 3.2. Load-Deflection Responses

**Figure 8:**
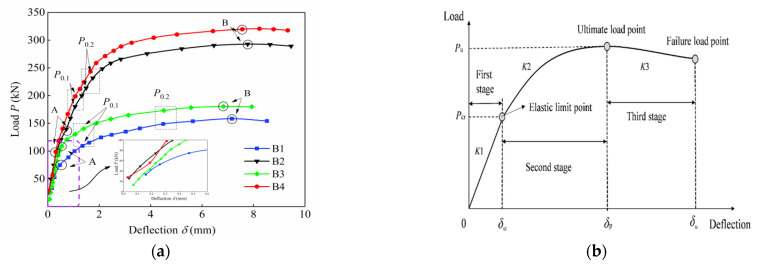
Load-deflection curves: (**a**) B1~B4, (**b**) typical load-deflection curve.

The deflections at the midspan of each specimen measured by the LVDT under diverse loading steps are shown in Figure 8; meanwhile, Figure 8b depicts the typical load-deflection curve of NSC and the fiber-reinforced concrete members under the flexural load, which include the descending segment as the load exerted the damage stage [38,39]. It can be seen from Figure 8a that: (1) The elastic working stage (before point A) was characterized by the cracking load *P*_cr_, and all specimens showed a quasi-linear load-deflection relationship and had not yet cracked. Therefore, the bridging effect of the steel fiber on the cracking resistance was not fully exploited. (2) The hardening stage was entered (between point A and point B) once the elastic-limit was broken. When the load was up to *P*_cr_, the first crack appeared at bottom of specimens, the tensile stress of UHPC matrix gradually transferred to steel fiber and tensile reinforcement together, and the stiffness thus decreased. Meanwhile, cracking resistance was sufficiently provided by steel fiber, and cracks developed sharply in number but relatively slowly in height and width. A non-linear correlation was dominant at this stage, and noticeable increases were found in the deflection followed a slight increment in load. (3) The third stage is the failure stage (after point B). After reaching the ultimate load *P*_u_, curves started to decrease slightly due to the local crushing of UHPC. Cracks developed sharply in width and extended along the height of specimens during this stage. It should be noted that vertical cracks distributed at the pure-bending section appeared rapidly and extended steadily. As the load further increased, the steel fiber was unable to bridge the crack and gradually exited the working state. In addition, for NRRU specimens, the cracking process acceleration was attributed to the reduction in the effective restraint ability when the reinforcement yielded, and thus the main visible crack was formed, and stiffness decreased remarkably. However, there was no distinct yield point for the high strength wire. Therefore, both the stress and stiffness decreased slowly until the high strength wire yielded, resulting in the smoother appearance of the load-deflection curves without an obvious turning point.

The main test results are collected in Table 6. The cracking load *P_c_*_r_ refers to the load when the first crack occurred, and at this moment the corresponding deflection is the cracking deflection *δ*_cr_. The ultimate load *P*_u_ presents the maximum load when the corresponding deflection reaches the peak deflection *δ*_u_. It should be noted that the test slab specimens have not been completely destroyed, as the test loading was manually controlled to prevent damage from improper operation; thus, no obvious descending segment was found, as compared to Figure 8b. The comparation of load capacity among two groups showed distinct differences. The corresponding load was obviously smaller in HSWRU specimens irrespective of the cracking load *P_c_*_r_, while the deflection showed a larger value instead. Moreover, for NRRU specimens, *P*_u_ was significantly increased by 85% compared to the HSWRU specimens, but *P_c_*_r_ exhibited a slight change, which only rose by 18%. Such enhancement was 33.55% for *P_c_*_r_ and 17.17% for *P*_u_, respectively, for specimen B3 with a 20% higher section-height as compared to B1. The main reason for this is that more steel fiber was provided in the higher section height to enhance the cracking resistance ability in the early stage. As the load further increased to the ultimate load, a sharp development in the crack width as well as height resulted in the fracture and pulling out of the steel fiber in UHPC, the enhancement of *P*_u_ was thereby greatly affected by the reinforcement at this moment. On the other hand, when the crack width reached 0.1 mm and 0.2 mm, the corresponding loads *P*_0.1_ and *P*_0.2_ accounted for 60%~85% of *P*_u_ but the deflections *δ*_0.1_ and *δ*_0.2_ for each specimen were less than 30% of the ultimate deflection *δ*_u_. This implies that the deformation of the UHPC members could be effectively controlled via the denser microstructure of the mixture, and the effect was better represented in HSWRU specimens attributed to the slower stiffness degeneration of the high-strength wire as well as stress. The larger stiffness and prolonged elastic period are expressly depicted in the Figure compared to the NRC members.

### 3.3. Strain Responses of Concrete

**Figure 9:**
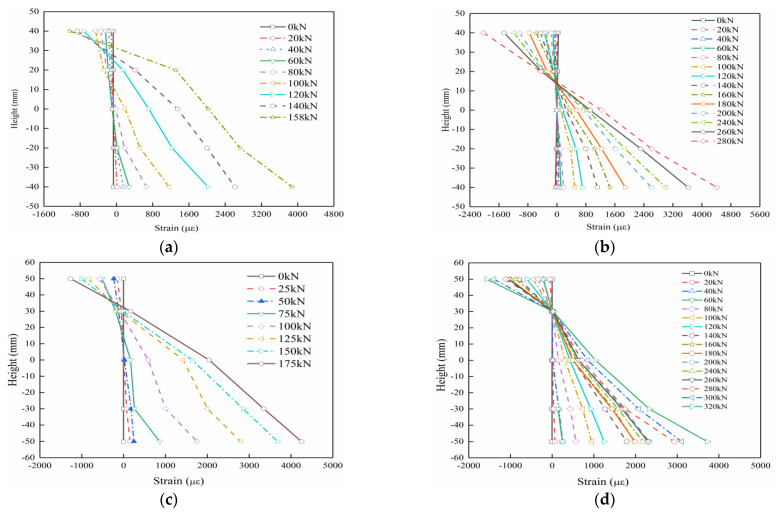
Strain profiles at the mid-span section of specimens: (**a**) B1, (**b**) B2, (**c**) B3, (**d**) B4.

Strain distribution along the section height of each slab specimen is plotted in Figure 9. The observed strain curves at various heights for each slab are believed to be basically linearly distributed, which verifies the flat section assumption.

## 4. Flexural and Ductility Calculation

Theoretical analysis is essential to further uncover the potential usage of reinforced UHPC specimens. Since the post-cracking strength was significantly improved in UHPC members [3,24], the contribution of concrete in the tension zone cannot be ignored when performing theoretical calculations in UHPC components [20]. However, the contribution of tensile stress was ignored in the current normal reinforced concrete (NRC) structural design code, and the tensile stress was considered triangularly distributed in the whole tensile zone at the cracking stage, which ignored the plastic development of UHPC. Furthermore, the energy dissipation and load capacity of UHPC members keeps rising as the deformation continues to grow [40], thus applying the conventional deformation ductility coefficient (*μ*_Δ_) for the ductility evaluation of UHPC components remains partial. With respect to the above considerations, the revised flexural calculation model and the energy ductility coefficient (*μ*_E_) is proposed for the theoretical analysis in the following section.

### 4.1. Basic Assumption

The following basic assumptions are made to calculate the flexural capacity and ductility coefficient of the slab specimens.

**Figure 10:**
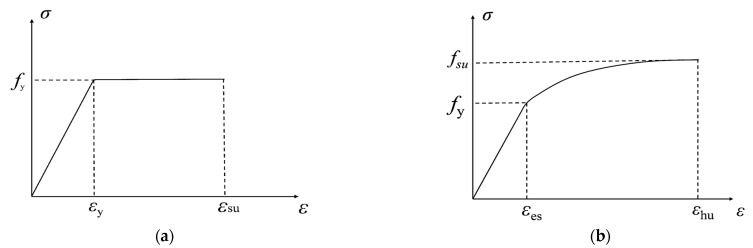
Stress–strain relationship curve of reinforcement: (**a**) HRB400, (**b**) high-strength wire.

(1) Considering the smaller strain in compressive zone and scarce cracks were observed on the top of the UHPC slabs, steel rebars arranged in the upper layer of the cross section do not yield when the ultimate load is reached, while rebars in tension zone yielded.
(2) Bond slip between UHPC material and steel bar is ignored, and the assumption regarding the flat section is applied to all test slabs.
(3) The constitutive relationship of the HRB400 steel bars adopts the ideal elastic-plastic constitutive relationship, and the resulting expression is described by Equation (3).
  (3) $$\left\{ \begin{array}{ll} \sigma_{s}=E_{s}\varepsilon_{s} & \;\;\varepsilon_{s}\leq \varepsilon_{y}\\ \sigma_{s}=f_{y} & \;\;\varepsilon_{y}\leq \varepsilon_{s}\leq \varepsilon_{su} \end{array}\right.$$
  where $f_{y}$ is the yield strength of the reinforcement, and $\sigma_{s}$ is the stress of steel rebar. $\varepsilon_{s}$ is corresponding strain of reinforcement. $\varepsilon_{y}$ and $\varepsilon_{su}$ are the yield strain and ultimate strain of reinforcement, respectively.
(4) The Lomberg model is used for the constitutive model of the high strength steel wire in this paper, can also be performed here as Equation (4).
  (4) $$\left\{ \begin{array}{ll} \varepsilon_{s}=\frac{\sigma_{s}}{E_{s}} & \;\;\varepsilon_{s}\leq \varepsilon_{es}\\ \varepsilon_{s}=\frac{\sigma_{s}}{E_{s}}+0.002{\left(\frac{\sigma_{s}}{f_{0.2}}\right)}^{13.5} & \;\;\varepsilon_{es}\leq \varepsilon_{s}\leq \varepsilon_{\mathrm{hu}} \end{array}\right.$$
  where $f_{0.2}$ is the conditional yield strength, once the plastic strain of the high strength steel wire reaches 0.2%. $\varepsilon_{es}$ and $\varepsilon_{hu}$ are the elastic strain limit and ultimate tensile strain of high-strength steel wire, respectively.
  The stress–strain relationship curve of HRB400 and high strength wire adopted in this test are plotted in Figure 10.
(5) The stress–strain curve of UHPC under compression adopts the relationship suggested by Xu et al. [41], for which the ascending section is simplified through the double broken line and the descending section is fitted by an oblique line suggested by Luo et al. [42], as shown in Equation (5).
  (5) $$\sigma_{c}=\left\{ \begin{array}{ll} E_{c}\varepsilon_{c} & \;\;\varepsilon_{c}<\varepsilon_{e}\\ \frac{0.2f_{c}}{\varepsilon_{p}-\varepsilon_{e}}(\varepsilon_{c}-\varepsilon_{e})+0.8f_{c} & \;\;\varepsilon_{e}<\varepsilon_{c}<\varepsilon_{p}\\ \beta_{1}\frac{f_{c}}{\varepsilon_{p}}\varepsilon_{c}+\beta_{2}f_{c} & \;\;\varepsilon_{p}<\varepsilon_{c}<\varepsilon_{cu} \end{array}\right.$$
  where $\sigma_{c}$ is the compressive stress corresponding to UHPC compressive strain $\varepsilon_{c}$. $E_{c}$ is the elastic modulus of UHPC, and $f_{c}$ is the axial compressive strength of UHPC. $\varepsilon_{e}$ is the elastic limit of UHPC. $\varepsilon_{p}$ is the peak strain of UHPC suggested by the previous literature $\varepsilon_{p}=(6.7264f_{c}+2460.9)\times 10^{-6}$ [43]. $\varepsilon_{cu}$ is the ultimate compressive strain of UHPC, as suggested by the literature [44], where $\varepsilon_{cu}=1.3\varepsilon_{p}$. $\beta_{1}$ and $\beta_{2}$ are fitting coefficients related to the content of steel fiber, and the value of *β*_1_ and *β*_2_ are taken as −0.392 and 1.392, respectively, as suggested by the literature [42].
(6) According to the conclusion of Hu et al. [45], the ascending section of UHPC tensile stress–strain curve can be regarded as an ideal elastic-plastic constitutive relationship, while the descending section adopts the formula obtained by Zhang et al. [46].
  (6) $$\sigma_{t}=\left\{ \begin{array}{ll} E_{c}\varepsilon_{t} & \;\;\varepsilon_{t}<\varepsilon_{t0}\\ f_{t} & \;\;\varepsilon_{t0}<\varepsilon_{t}<\varepsilon_{tp}\\ \frac{f_{t}}{{\left(1+(\varepsilon_{t}-\varepsilon_{tp})l_{c}/w_{p}\right)}^{p}} & \;\;\varepsilon_{t}>\varepsilon_{tp} \end{array}\right.$$
  where $\sigma_{t}$ is the corresponding tensile stress of UHPC, $\varepsilon_{t0}$ is the tensile strain of UHPC when the tensile stress $f_{t}$ is reached. $\varepsilon_{tp}$ is the peak tensile strain of UHPC, the empirical formula is given by Guo et al. [43], i.e., $\varepsilon_{tp}=0.3/l_{c}+\varepsilon_{t0}$. $l_{c}$ is the characteristic length, as the rectangular section can be replaced as $l_{c}=2/3h$, and *h* is the section height. p is the test fitting parameter, and $w_{p}$ is the crack width when stress drops to $2^{-p}f_{c}$, the value of which is obtained by applying the test results, $w_{p}=1.00$ and p=0.95, respectively.

**Figure 11:**
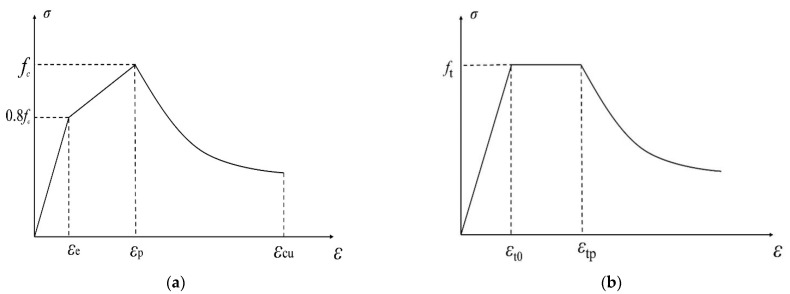
Stress–strain relationship curve of UHPC in compression and tension: (**a**) Compression, (**b**) tension.

Figure 11 shows the stress–strain relationship curve of UHPC in compression and tension.

### 4.2. Cracking Moment Capacity

**Figure 12:**
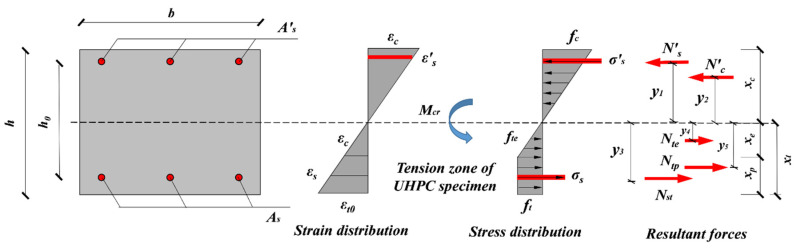
Calculation models of cracking moment.

Owing to the higher bond strength between the steel fiber and concrete matrix; improved plastic development degree and higher ultimate tensile strain are thereby obtained for UHPC members at the cracking stage [20,47], resulting in the irregularly distributed tensile stress in the tensile area that is distant from the neutral axis ($f_{t}$), while the stress at the near part appeared triangular instead ($f_{t}{}_{e}$). To simplify the calculation, tensile stress distributed at the plastic region is regarded as rectangularly distributed and the resultant forces are calculated by $N_{t}{}_{e}=E_{c}\varepsilon_{t}bx_{e}$ and $N_{\mathrm{tp}}=E_{c}\varepsilon_{t0}bx_{p}$, respectively, where *x*_p_ is the corresponding height of $f_{t}$. Moreover, the resultant force of concrete in compressive zone is expressed as $N\prime_{c}=E_{c}\varepsilon_{t0}bx_{c}$, and for reinforcement the resultant force is calculated by $N\prime_{s}=N_{s}=E_{s}\varepsilon_{s}A_{s}$, since the steel rebar does not yield at this point. The calculation models are shown in Figure 12.

Based on the flat section assumption, the strain and stress of UHPC and the reinforcement can be calculated by using the geometric relationship as follows:

(7) $$\varepsilon_{s}^{\prime }=\varepsilon_{t0}\frac{\left(x_{c}-a\prime_{s}\right)}{x_{e}}=\varepsilon_{t0}\frac{\left(x_{c}-a\prime_{s}\right)}{(1-\lambda )x_{t}}=\varepsilon_{t0}\frac{\left(x_{c}-a\prime_{s}\right)}{\left(1-\lambda )(h-x_{c}\right)}$$

(8) $$\varepsilon_{c}=\varepsilon_{t0}\frac{x_{c}}{x_{e}}=\varepsilon_{t0}\frac{x_{c}}{\left(1-\lambda )(h-x_{c}\right)}$$

(9) $$\varepsilon_{s}=\varepsilon_{t0}\frac{\left(x_{t}-a_{s}\right)}{x_{e}}=\varepsilon_{t0}\frac{\left(x_{t}-a_{s}\right)}{(1-\lambda )x_{t}}=\varepsilon_{t0}\frac{\left(h-x_{c}-a_{s}\right)}{\left(1-\lambda )(h-x_{c}\right)}$$

where $x_{c}$ is the compression height and λ is the plastic development coefficient, as presented as below:

(10) $$\lambda =\frac{x_{p}}{x_{t}}$$

According to the equilibrium condition $\Sigma F_{x}=0$:

(11) $$N\prime_{s}+N\prime_{c}=N_{st}+N_{te}+N_{tp}$$

Replacing the resultant force from the expression of stress and strain, the equilibrium equation above is expressed as:

(12) $$\frac{E_{s}}{E_{c}}f_{t}\frac{\left(x_{c}-a_{s}\right)}{\left(1-\lambda )(h-x_{c}\right)}{A_{s}}^{\prime }+\frac{1}{2}\frac{bf_{t}x_{c}^{2}}{\left(1-\lambda )(h-x_{c}\right)}=\frac{E_{s}}{E_{c}}f_{t}\frac{\left(h-x_{c}-a_{s}\right)}{\left(1-\lambda )(h-x_{c}\right)}A_{s}+\frac{1}{2}f_{t}b(1+\lambda )(h-x_{c})$$

where ${A_{s}}^{\prime }$ and $A_{s}$ represent the area of reinforcement in the compression and tension zone, respectively.

Based on the equilibrium condition, the cracking moment is as calculated below $\Sigma M_{\mathrm{cr}}=0$.

(13) $$M_{cr}=N\prime_{s}y_{1}+N\prime_{c}y_{2}+N_{st}y_{3}+N_{te}y_{4}+N_{tp}y_{5}$$

where $y_{1}$ and $y_{5}$ are the corresponding distances from the resultant forces $N\prime_{s},N\prime_{c},N_{st},N_{te}$ and $N_{tp}$ to the neutral axis, respectively, which can be calculated by the following formulas:

(14) $$\{\begin{array}{c} y_{1}{=x}_{c}-a\prime_{s}\\ y_{2}=\frac{2}{3}x_{c}\\ y_{3}=h-x_{c}-a_{s}\\ y_{4}=\frac{1}{3}(h-x_{c})(2+\lambda )\\ y_{5}=(1-\frac{\lambda }{2})(h-x_{c}) \end{array}$$

Substituting Equations (7)–(9) and Equation (14) to Equation (13), the final calculation formula for cracking moment *M*_cr_ is given as:

(15) $$\begin{array}{cc} M_{cr} & =N\prime_{s}y_{1}+N\prime_{c}y_{2}+N_{st}y_{3}+N_{te}y_{4}+N_{tp}y_{5}\\ & =\frac{E_{s}}{E_{c}}f_{t}\frac{\left(x_{c}-a_{s}\right)}{\left(1-\lambda )(h-x_{c}\right)}{A_{s}}^{\prime }(x_{c}-a_{s})+\frac{1}{3}\frac{bf_{t}x_{c}^{3}}{\left(1-\lambda )(h-x_{c}\right)}\frac{E_{s}}{E_{c}}f_{t}\\ & +\frac{\left(h-x_{c}-a_{s}\right)}{\left(1-\lambda )(h-x_{c}\right)}A_{s}+\frac{\lambda }{3}f_{t}b(1-\lambda )(h-x_{c}{)}^{2}+f_{t}b(1-\frac{\lambda }{2})(h-x_{c}) \end{array}$$

### 4.3. Ultimate Moment Capacity

**Figure 13:**
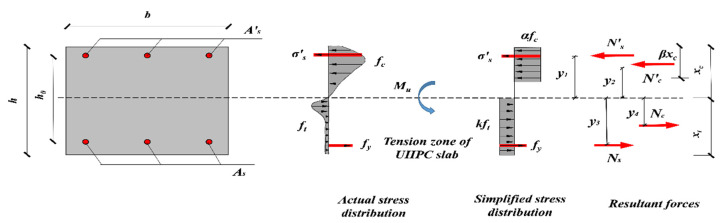
Calculation models of ultimate moment.

The analytical models for ultimate flexural capacity calculation are shown in Figure 13. As previously declared, reinforcement in tension yielded ($f_{y}$), while reinforcement in compression was not ($\sigma_{s}^{\prime }$). Cracks were relatively small and evenly distributed in the tensile zone when reaching the ultimate stage, the bridge effect of steel fiber was thus maintained [7]. Moreover, the smaller height of the compressive zone of UHPC specimens resulted in the higher tensile height, and the cracking strength ($f_{t}$) of UHPC is thereby assumed to be maintained and the equivalent rectangular stress distribution diagram is used in the tensile zone. The equivalent compressive stress and tensile stress are changed into $\alpha f_{c}$ and $kf_{t}$ with the heights $\beta x_{c}$ and $x_{t}$, respectively. Where α, β and k represent the reduction coefficient.

According to the principle of equivalence, the compressive resultant force of concrete ($N\prime_{c}$) is calculated by:

(16) $$N\prime_{c}=\int_{0}^{x_{c}}\sigma_{c}(\varepsilon_{c})bdy$$

The corresponding distance from the neutral axis *y*_1_ is expressed by:

(17) $$y_{1}=\frac{\int_{0}^{x_{c}}\sigma_{c}(\varepsilon_{c})bydy}{N\prime_{c}}$$

According to the geometric relationship:

(18) $$\varepsilon_{y}=\frac{y}{x_{c}}\varepsilon_{cu}$$

where $\varepsilon_{y}$ is the strain at the position that departs from the neutral axis, y is the corresponding distance.

Taking Equation (18) into Equation (16) and (17), $N\prime_{c}$ and $y_{1}$ can be written as:

(19) $$N\prime_{c}=\int_{0}^{\varepsilon_{cu}}\sigma_{c}(\varepsilon_{c})d\varepsilon_{c}$$

(20) $$y_{1}=\frac{b{\left(\frac{x_{c}}{\varepsilon_{cu}}\right)}^{2}\int_{0}^{\varepsilon_{cu}}\sigma_{c}(\varepsilon_{c})\varepsilon_{c}d\varepsilon_{c}}{\frac{bx_{c}}{\varepsilon_{cu}}\int_{0}^{\varepsilon_{cu}}\sigma_{c}(\varepsilon_{c})d\varepsilon_{c}}\;$$

To simplify the calculation process, replacing $k_{1}=\frac{N\prime_{cu}}{f_{c}\varepsilon_{cu}}$ and $k_{2}=\frac{y_{cu}}{\varepsilon_{cu}}$, where $N\prime_{cu}$ and $y_{cu}$ are calculated as follows:

(21) $$N\prime_{cu}=\int_{0}^{\varepsilon_{cu}}\sigma_{c}(\varepsilon_{c})d\varepsilon_{c}$$

(22) $$y_{cu}=\frac{\int_{0}^{\varepsilon_{cu}}\sigma_{c}(\varepsilon_{c})\varepsilon_{c}d\varepsilon_{c}}{\int_{0}^{\varepsilon_{cu}}\sigma_{c}(\varepsilon_{c})d\varepsilon_{c}}\;$$

Then, the resultant force $N\prime_{c}$ and corresponding distance $y_{1}$ can be extended as:

(23) $$N\prime_{c}=\alpha f_{c}b\beta x_{c}=k_{1}f_{c}bx_{c}$$

(24) $$y_{1}=k_{2}x_{c}\;$$

Based on the equivalence principle, the following relationships are easily obtained:

(25) $$\alpha \beta =k_{1}$$

(26) $$\frac{1}{2}\beta x_{c}=x_{c}-y_{1}\;$$

Combining the Equations (25) and (26), the solution to α and β is given below:

(27) $$\alpha =\frac{k_{1}}{\beta }=\frac{k_{1}}{2(1-k_{2})}$$

Since the values of $N\prime_{cu}$ and $y_{\mathrm{cu}}$ are determined by material properties, the values $k_{1}=0.62$ and $k_{2}=0.64$ are gained from the previously mentioned constitutive relationship. Substituting $k_{1}$ and $k_{2}$ into Equation (27), the values of α=0.86 and β=0.72 are obtained, respectively. Then, the resultant force of concrete in compression is set as $N_{c}^{\prime }=\frac{1}{2}\alpha f_{c}\beta bx_{c}{}^{2}$ and $N_{c}=\frac{1}{2}b{\left(h-x_{c}\right)}^{2}kf_{t}$, and the resultant forces for reinforcement in compression and tension are calculated as $N_{s}^{\prime }=E_{s}\varepsilon_{s}^{\prime }A_{s}^{\prime }$, and $N_{s}=f_{y}A_{s}$, respectively.

Considering the equilibrium condition $\Sigma F_{x}=0$*:*

(28) $$N\prime_{s}+N\prime_{c}=N_{c}+N_{s}$$

The ultimate moment $M_{u}$ can be established by a taking moment of force of the neutral axis ΣM=0*:*

(29) $$M_{u}=N\prime_{s}y_{1}+N\prime_{c}+N_{s}y_{3}+N_{c}y_{4}$$

where $y_{1}$ − $y_{4}$ are the corresponding distance from the resultant forces $N\prime_{s},N\prime_{c},N_{s}$, and $N_{c}$ on the neutral axis, respectively, which are given by following formulas:

(30) $$\{\begin{array}{c} y_{1}=x_{c}-a\prime_{s}\\ y_{2}=\frac{1}{2}x_{c}\\ y_{3}=h-x_{c}-a_{s}\\ y_{4}=\frac{1}{2}(h-x_{c}) \end{array}$$

Decomposing Equation (28) and (29) expressed by strain and stress, the predicted ultimate moment $M_{u}$ can be calculated through the following formulas:

(31) $$\frac{E_{s}\varepsilon_{cu}(x_{c}-a_{s}^{\prime })}{x_{c}}A_{s}^{\prime }+\alpha f_{c}b\beta x_{c}=f_{y}A_{s}+(h-x_{c})bkf_{t}$$

(32) $$M_{u}=\frac{E_{s}\varepsilon_{cu}{\left(x_{c}-a_{s}^{\prime }\right)}^{2}}{x_{c}}A_{s}^{\prime }+\frac{1}{2}\alpha f_{c}\beta x_{c}{}^{2}b+f_{y}A_{s}(h-x_{c}-a_{s})+\frac{1}{2}b{\left(h-x_{c}\right)}^{2}kf_{t}$$

### 4.4. Ductility Coefficient

**Figure 14:**
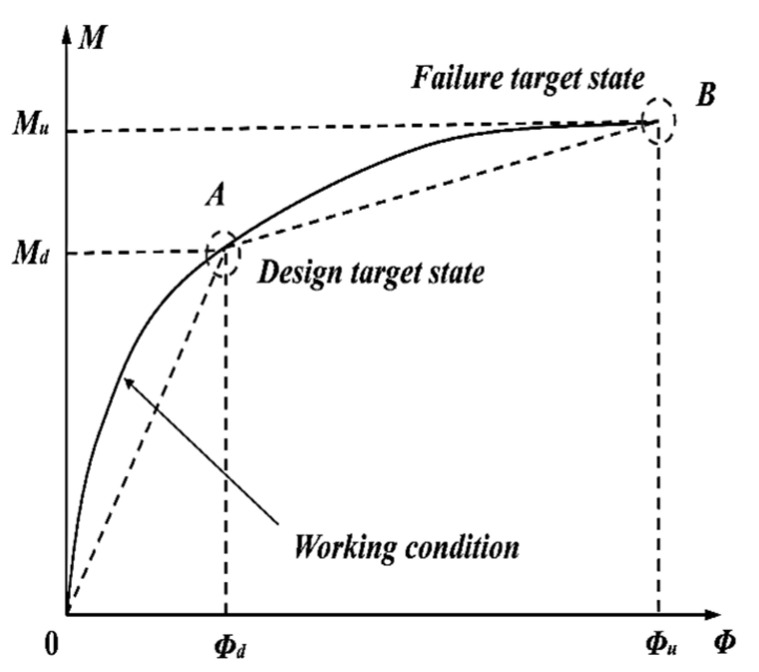
Two characteristic states on the moment–curvature curve.

**Figure 15:**
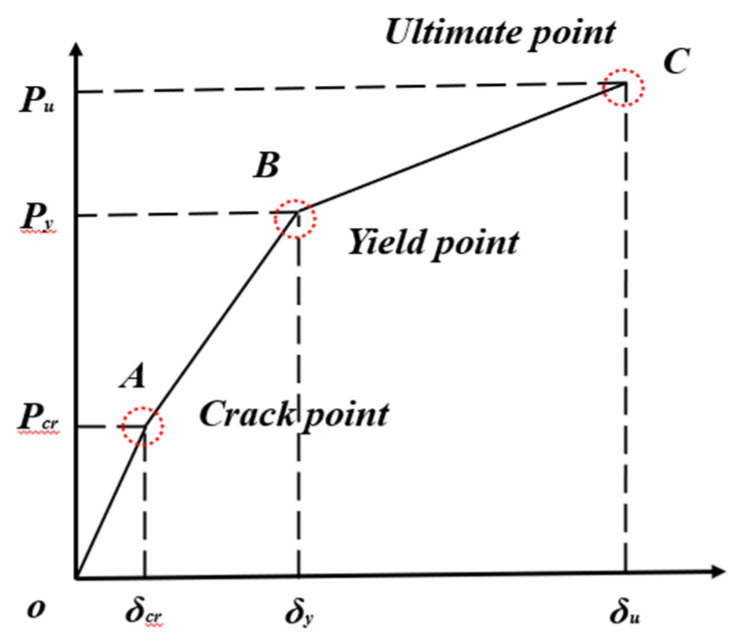
Simplified ductility coefficient calculation model.

As previously stated, since $\mu_{\Delta }$ cannot reflect the ductility performance of UHPC components reasonably, $\mu_{E}$ is thereby introduced to comprehensively evaluate the ductility of UHPC members, which is expressed as $\mu_{E}=\frac{J_{u}}{J_{y}}$, where *J*_y_ and *J*_u_ present the yield and the ultimate deformation energy of specimens, as depicted in Figure 14 [48]. Meanwhile, the three-line model of the load-deflection curve is adopted for ductility calculations, as shown in Figure 15. The theoretical energy ductility coefficient ($\mu_{\mathrm{ES}}$) is calculated by $\mu_{\mathrm{ES}}=\frac{S_{\mathrm{OABC}}}{S_{\mathrm{OAB}}}$, where *S*_OABC_ and *S*_OAB_ are the areas enclosed by the point of ultimate load and yield load on the broken line, respectively.

### 4.5. Validation

The comparation between experimental and theoretical results of flexural capacity ($M_{\mathrm{cr}}^{c}$ and $M_{u}^{c}$) is listed in Table 7, where λ and k were obtained by substituting the test results into the proposed formulas. It is apparent that both the experimental and theoretical results of the tested specimens are in good agreement. The average ratios of experimental and theoretical capacity are 1.08 and 1.06, respectively, at the cracking and ultimate stage, with the corresponding variation coefficient 0.0077 and 0.0071, respectively. The average value of $M_{\mathrm{cr}}^{c}$ and $M_{u}^{c}$ is around 10% smaller than the average test value $M_{\mathrm{cr}}^{t}$ and $M_{\mathrm{cr}}^{t}$, which are similar to the results presented in the studies [18,22]. This observed reduction indicates that the proposed equations are qualified with a securely flexural capacity prediction. In addition, the introduced plastic development coefficient λ ranges from 0.41 to 0.48, showing that the plasticity in the tension zone was developed to some extent when the specimen cracked. This can be explained by the relatively smaller upward movement of the neutral axis, thereby resulting in the higher degree of the plastic region. However, due to the deficiency in the number of specimens, a further study should be carried out to apply the investigation results on more and larger-scale-designed UHPC slabs in order to obtain more verifiable information.

**Figure 16:**
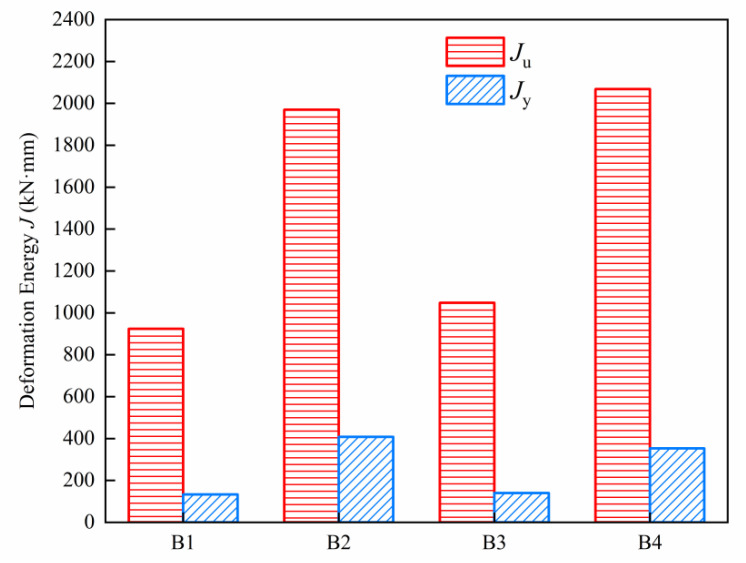
Deformation energy of test slabs.

The deformation energy (*J*) of the specimens is shown in Figure 16, and the values of $\mu_{E}$ and $\mu_{ES}$ are summarized in Table 8. The maximum deviation is no more than 7% between $\mu_{E}$ and $\mu_{ES}$ with the variation coefficient at 0.032, indicating that the theoretical results originated from the simplified model has enough reliability, and previous assumptions are thereby properly made. As evident in the table, for HSWRU specimens, $\mu_{E}$ is 6.95 and 7.49, respectively, which is higher than NRRU specimens with the values of 4.82 and 5.96. The deformation ductility coefficient $\mu_{\Delta }$ is higher than the tested specimens in the studies [17] and [26], and the excellent ductility performance of the designed specimens is thereby substantiated. In particular, a nearly 30% promotion ($D_{p}$) is noted by comparing $\mu_{E}$ to the deformation ductility coefficient $\mu_{\Delta }$, and it is more apparent for HSWRU specimens with a remarkable increase in value of 33.65% and 34.47%, respectively. This finding implies that although the deformation ductility coefficient $\mu_{\Delta }$ gives a higher safety consideration, the considerable mechanical properties of UHPC specimens may not be appropriately evaluated, and $\mu_{E}$ is notably increased in contrast to $\mu_{\Delta }$ due to the better cooperative working ability among HSW and UHPC.

##  5. Parametric Analysis

In order to further reveal the influencing factors on the flexural response and ductility, the parametric analysis was carried out in the factors of the reinforcement ratio (*ρ*), the strength of reinforcement (*f*_y_) and the section height (*h*). Compared with the experimental layout, the variation range of different parameters was broadened in UHPC specimens to obtain more reliable conclusions. Typical components B1 and B2 were chosen to perform the parametric analysis, including the main parameters as follows: *l_0_* = 600 mm, *b* = 320 mm, *a_s_* = *a*′*_s_* = 10 mm, *h* = 100 mm, *f_y_* = 1270 Mpa and *ρ* = 0.43% for B1, while for B2 was *l_0_* = 600 mm, *b* = 320 mm, *a_s_* = *a*′*_s_* = 10 mm, *h* = 120 mm, *f_y_* = 400 Mpa and *ρ* = 2.94%, where *l_0_* is the clear span of the test slab. Where *J*_y-N_, *J*_y-H_, *J*_u-N_ and *J*_u-H_ present the yield deformation energy *J*_y_ and the ultimate deformation energy *J*_u_ for NRRU and HSWRU specimens, respectively, *P*_y-N_, *P*_y-H_, *P*_u-N_ and *P*_u-H_ are the corresponding load capacities.

**Figure 17:**
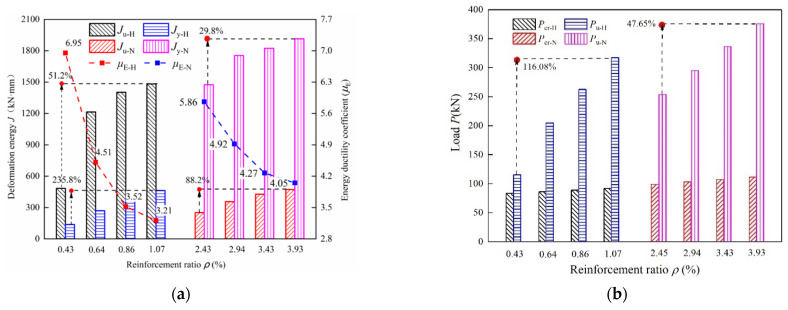

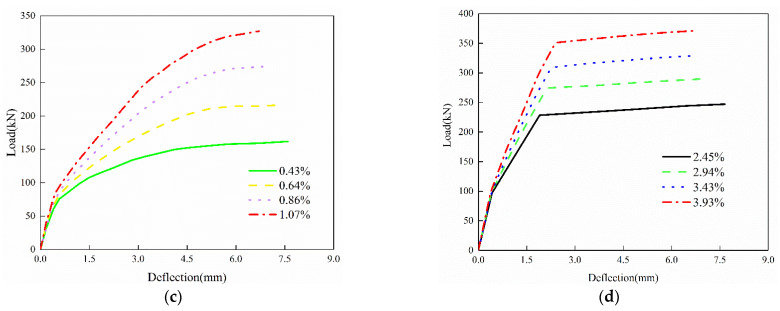
Effect of the reinforcement ratio on the energy ductility coefficient and load-deflection curves of specimens: (**a**) Ductility coefficient and deformation energy, (**b**) bearing capacity, (**c**) effect of ρ on load-deflection curve for HSWRU specimens, (**d**) effect of ρ on load-deflection curve for NRRU specimens.

Figure 17 compares the flexural behavior and load-deflection curves for *ρ* = 2.45%~3.93% in the NRRU specimens and *ρ* = 0.43%~1.07% in HSWRU specimens. The relationship of $\mu_{E}$ and *J* versus the reinforcement ratios *ρ* are depicted in Figure 17a. Overall, a noticeable reduction is found for all specimens with the increase of *ρ*, implying that the reinforcement ratio greatly effects the ductility of specimens. As *ρ* increases from 0.43% to 1.07%, *J*_y-H_ significantly increases by 235.8% while *J*_u-H_ increases by 51.2%, leading *μ*_E-H_ to decrease from 6.95 to 3.21. It can be inferred that although the enhancement in reinforcement ratio *ρ* is beneficial for the improvement of *P*_y_ and *P*_u_, the ductility is significantly weakened instead. Likewise, when *ρ* raises from 2.45% to 3.93%, *μ*_E-N_ drops from 5.86 to 4.05 for NRRU slabs. The influence of the reinforcement ratio on flexural load capacity is illustrated in Figure 17b. As is clearly shown in the Figure, when *ρ* increases for both HSWRU and NRRU specimens, the ultimate load increases by 47.65% in *P*_u-N_ and 116.08% in *P*_u-H_, respectively. Whereas increment in cracking load *P*_cr-H_ and *P*_cr-N_ are smaller than 15%. It can be concisely supposed that the increase of *ρ* does not provide the cracking load *P*_cr_ with a significant increment. This phenomenon can be attributed to the fact that before the cracking stage, external load is mainly born by the concrete and the reinforcement has little effect on capacity enhancement. Once the ultimate stage is approached, stress is initially sustained by the steel fiber and concrete transferred to the longitudinal reinforcement, which significantly influences the ultimate capacity. With respect to the load-deflection curves, the comparations plotted in Figure 17c,d indicate that load-deflection curves for HSWRU specimens grow more rapidly with non-linear condition as *ρ* increases. By contrast, the NRRU specimens tend to increase in mild steps, in which the increment is basically in line with the former step, leading a higher increase in the amplitude of deformation energy in HSWRU specimens as compared to NRRU specimens.

**Figure 18:**
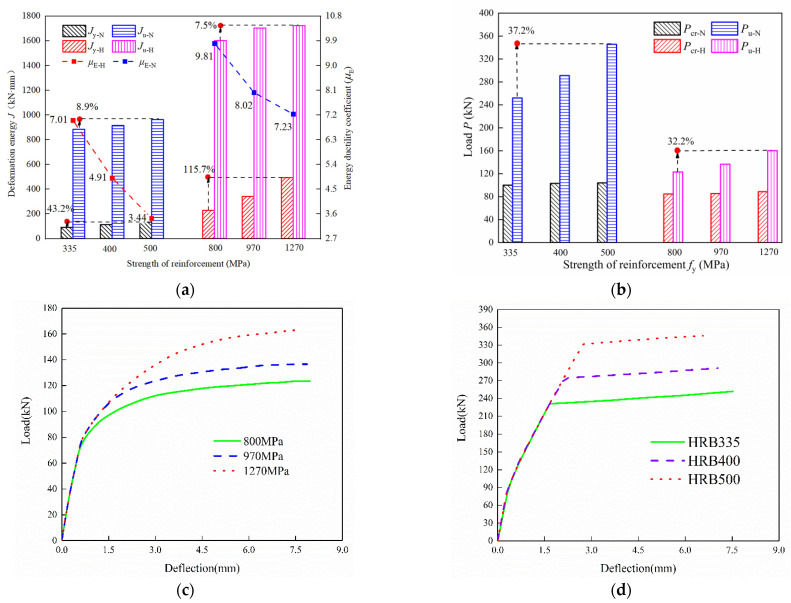
Effect of strength of reinforcement on the energy ductility coefficient and load-deflection curves of specimens: (**a**) Ductility coefficient and deformation energy, (**b**) bearing capacity, (**c**) effect of *f*_y_ on load-deflection curve for HSWRU specimens, (**d**) effect of *f*_y_ on load-deflection curve for NRRU specimens.

Figure 18 compares the flexural behavior and load-deflection curves of *f*_y_ = 335~500 MPa for NRRU specimens and *f*_y_ = 800~1270 MPa for HSWRU specimens. Figure 18a depicts the impact of the reinforcement strength on ductility coefficient and energy deformation. There is a remarkable increase in the yield deformation energy for both HSWRU and NRRU specimens when *f*_y_ changing from 335 MPa to 500 MPa in NRRU and from 800 MPa to 1270 MPa in HSWRU specimens, where *J*_y-H_ increases by 115.7% and *J*_y-N_ by 43.2%. However, there is almost no increase in *J*_u_, resulting in the 50.9% and 30.43% decrease in *μ*_E-N_ and *μ*_E-H_. To be noted, in compliance with the slower stress reduction and the smaller stiffness degeneration for HSW, the increment in *J*_y-H_ is found to be more than twice as compared to *J*_y-N_, indicating that the material properties of HSW are better exploited in UHPC. Figure 18b points out that *P*_u-H_ and *P*_u-N_ increase by 37.2% and 32.2%, respectively, as *f*_y_ increases but cracking capacity *P*_cr_ remains almost the same. This observation is consistent with the description mentioned above: the reinforcement dominants during the ultimate stage while it is not at cracking capacity. It is apparent in Figure 18c,d that when increasing *f*_y_, the load-deflection curves steadily go upward for both HSWRU and NRRU. The explanation for such behavior is that within a certain range, the beneficial degree of reinforcement ratio is greater than the strength of reinforcement on flexural capacity enhancement. Moreover, it is worth noting that *μ*_E-N_ sharply decreases by around 50% when *f*_y_ ranges from 335 MPa to 500 MPa. In the case of HSWRU specimens, reduction in *μ*_E-N_ is less than 30% when *f*_y_ increases from 800 MPa to 1270 MPa. The recommendation of high-strength reinforcement in UHPC components is thus further supported.

**Figure 19:**
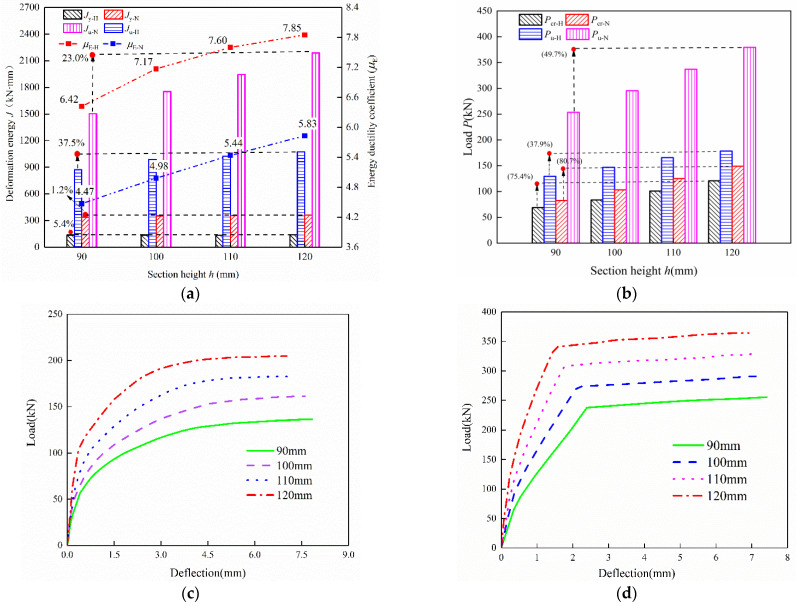
Effect of section height on the energy ductility coefficient and load-deflection curves of specimens: (**a**) Ductility coefficient and deformation energy, (**b**) bearing capacity, (**c**) effect of *h* on load-deflection curve for HSWRU specimens, (**d**) effect of *h* on load-deflection curve for NRRU specimens.

Figure 19 compares the flexural behavior and load-deflection curves for *h* = 90~120 mm. Figure 19a evidently shows that the section height significantly impacts the ductility coefficient. When *h* increasing from 90 mm to 120 mm, the ductility coefficient $\mu_{E-H}$ increases from 6.42 to 7.85 and $\mu_{E-N}$ increases from 4.47 to 5.83, respectively. Moreover, the deformation energy *J*_u-H_ and *J*_u-N_ increases by 37.52% and 23.0%, respectively, while *J*_y_ only increases by approximately 5%. This is because the stiffness of the UHPC specimen is improved with the increase of section height, resulting in the reduction of compressive height and thereby both the yield displacement *δ*_y_ and ultimate displacement *δ*_u_ decrease, while *δ*_y_ decreases more obviously, as shown in Figure 19c,d. As for the flexural capacity, Figure 19b illustrates that there is a noticeable increase in both *P*_cr_ and *P*_u_ as *h* ascends from 90 mm to 120 mm, and this trend is more significant in *P*_cr_, which increases by 75.44% in *P*_cr-H_ and 80.74% in *P*_cr-N_. Furthermore, the ultimate load of *P*_u-H_ and *P*_u-N_ increased by 37.87% and 49.7%, respectively. In general, the higher section height is highlighted for both the ductility and flexural capacity augment, especially for the cracking resistance consideration.

## 6. Conclusions and recommendation

This paper presents an experimental study on the structural response of HSWRU and NRRU specimens. The main conclusions were drawn as follows:

(1) The cracking and failure modes indicate that reinforced UHPC specimens exhibit great cracking resistance ability. The cracking development process was more sufficient in NRRU specimens due to the higher reinforcement ratio.
(2) The bending process mainly consisted of elasticity, hardening and damage stages, and HSWRU specimens are longer in the elastic stage and smaller in stiffness change compared to NRRU specimens; thus, the load-deflection curve presents as smoother. The excellent mechanical properties of UHPC are better used for co-operating with high strength wire.
(3) The theoretical results on cracking and the ultimate capacity of UHPC slabs match well with the relevant experimental results, which are around 10% smaller than test values, indicating that they can be applied for the flexural capacity prediction of reinforced UHPC slabs with safety reservations. Tests performed on more specimens of a larger scale suggest the further verification of the applicability of the proposed formulas.
(4) Good agreement between theoretical and test results substantiates the precision of the issued simplified model for ductility calculation. Moreover, it was to be found too conservative to evaluate the ductility performance of UHPC components in the conventional deformation ductility coefficient, especially for HSWRU specimens.
(5) Enhancement in section height is beneficial for both flexural capacity and ductility, especially for NRRU specimens. The reinforcement ratio and strength of the steel rebar are considerable for the improvement of the ultimate capacity, but indistinctive regarding the cracking load enhancement. Moreover, those two factors weaken the ductility and ultimate deflection of HSWRU specimens; thereby, the reasonable reinforcement ratio should be addressed during the design process of this type of specimen.

## Figures and Tables

**Table 1:** Key parameters of specimens.

Specimen	*B* (mm)	*H* (mm)	Longitudinal Reinforcement		Reinforcement Number	*f_y_* (MPa)	*ρ* (%)
			Top	Bottom			
B1	320	100	HSW	HSW	7 × 2	1270	0.86
B2	320	100	HRB400	HRB400	3 × 2	400	5.89
B3	320	120	HSW	HSW	7 × 2	1270	0.72
B4	320	120	HRB400	HRB400	3 × 2	400	4.91

**Table 2:** Mix proportions of UHPC.

Component	Cement	Silica Fume	Quartz Sand	Quartz Powder	Slag Powder	Water	Fly ash	Water Reducer
Mass ratio	1.0	0.2	1.1	0.1	0.15	0.22	0.1	0.02

**Table 3:** Properties of steel fiber.

	Tensile Strength (MPa)	Length *l* (mm)	Diameter *D* (mm)	*l*/*d*	Modulus of Elasticity (GPa)
Steel fiber	>2000 MPa	10	0.2	50	210

**Table 4:** Mechanical properties of different steels.

Materials	Diameter *d*(mm)	Yield Strength *f_y_* (MPa)	Ultimate Strength *f_su_* (MPa)	Modulus of Elasticity *E*_s_ (GPa)
HBR400	20	452	590	201
High strength wire	5	1121	1316	205

**Table 5:** Material properties of UHPC mixture.

Marks	*f*_c_ (MPa)	Mean	*E* (GPa)	Mean	*S*	*f_fl_* (MPa)	Mean	*f_t_* (MPa)	Mean
T1	123.9		47.08			15.16		7.82	
T2	130.8	128.1	47.46	46.91	0.46	14.59	15.32	7.44	7.51
T3	129.9		46.18			16.23		7.27	

**Table 6:** Summary of main test results.

Specimen	*P*_cr_ (kN)	*δ*_cr_ (mm)	*P*_0.1_ (kN)	*δ*_0.1_ (mm)	*P*_0.2_ (kN)	*δ*_0.2_ (mm)	*P*_u_ (kN)	*δ*_u_ (mm)
B1	88.61	0.80	109.43	1.35	125.35	2.48	158.34	8.53
B2	102.45	0.46	184.28	1.11	213.62	1.51	293.68	9.46
B3	120.94	0.74	135.23	1.39	153.72	2.45	180.46	8.08
B4	150.74	0.51	203.56	1.45	244.73	1.88	322.49	9.27

**Table 7:** Comparison between calculated and tested ultimate load values.

Specimen	*λ*	$M_{cr}^{t}\;(\mathrm{kN}\cdot m)$	$M_{\mathrm{cr}}^{c}\;(\mathrm{kN}\cdot m)$	$\frac{M_{cr}^{t}}{M_{cr}^{c}}$	Mean	*k*	$M_{u}^{t}\;(\mathrm{kN}\cdot m)$	$M_{u}^{c}\;(\mathrm{kN}\cdot m)$	$\frac{M_{u}^{t}}{M_{u}^{c}}$	Mean
B1	0.48	8.86	8.22	1.08	1.08	0.30	15.83	14.35	1.05	1.06
B2	0.41	10.24	9.78	1.07		0.25	29.37	28.14	1.04	
B3	0.44	12.09	10.88	1.09		0.28	17.32	16.07	1.08	
B4	0.43	15.07	14.39	1.05		0.27	32.25	30.44	1.06	

**Table 8:** Comparison of calculated and tested ductility coefficient values.

Specimen	Δ_y_	Δ_u_	*μ*_Δ_	*μ*_E_	*μ*_ES_	*D*_p_ (%)	Mean	*μ*_E_/*μ*_ES_	Mean	Var
B1	1.64	8.53	5.20	6.95	6.76	33.65	27.95%	1.03	1.02	0.0011
B2	2.36	9.46	4.01	4.82	4.92	20.20		0.98		
B3	1.41	7.86	5.57	7.49	7.02	34.47		1.07		
B4	2.13	9.44	4.43	5.47	5.77	23.47		1.01		

## Data Availability

Not applicable.

## References

[1] Hakeem I.Y., Rahman M.K., Althoey F. (2022). Experimental Investigation of Hybrid Beams Utilizing Ultra-High Performance Concrete (UHPC) as Tension Reinforcement. Materials.

[2] Paredes J.A., Galvez J.C., Enfedaque A., Alberti M.G. (2021). Matrix Optimization of Ultra High Performance Concrete for Improving Strength and Durability. Materials.

[3] Ferdosian I., Camoes A. (2021). Mechanical performance and post-cracking behavior of self-compacting steel-fiber reinforced eco-efficient ultra-high performance concrete. Cem. Concr. Compos..

[4] Dai J., Huang B., Shah S. (2021). Recent advances in strain-hardening UHPC with synthetic fibers. J. Compos. Sci..

[5] Du J., Meng W., Khayat K.H., Bao Y., Guo W., Lyu Z., Abu-obeidah A., Nassif H., Wang H. (2021). New development of ultra-high-performance concrete (UHPC). Compos. Part. B-Eng..

[6] Liao J., Yang K., Zeng J., Quach W., Ye Y., Zhang L. (2021). Compressive behavior of FRP-confined ultra-high performance concrete (UHPC) in circular columns. Eng. Struct..

[7] Bajaber M.A., Hakeem I.Y. (2021). UHPC evolution, development, and utilization in construction: A review. J. Mater. Res. Technol..

[8] Shi G., Ban H., Shi Y., Wang Y. (2013). Overview of research progress for high strength steel structures. Eng. Mech..

[9] Sun B., Wang D., Lu X., Wan D., Ponge D., Zhang X. (2021). Current challenges and opportunities toward understanding hydrogen embrittlement mechanisms in advanced high-strength steels: A review. Acta. Metall. Sin-Engl..

[10] Ban H., Shi G. (2018). A review of research on high-strength steel structures. Proc. Inst. Civ. Eng. -Struct. Build..

[11] Ban H., Shi G., Shi Y., Wang Y. (2012). Overall buckling behavior of 460 MPa high strength steel columns: Experimental investigation and design method. J. Constr. Steel. Res..

[12] Ban H., Shi G., Shi Y., Wang Y. (2013). Column buckling test of 420 MPa high strength steel single equal angles. Int. J. Struct. Stab. Dy..

[13] Ban H., Shi G., Shi Y., Wang Y. (2012). Residual stress tests of high-strength steel equal angles. J. Struct. Eng.-ASCE.

[14] Chiew S., Zhao M., Lee C. (2015). Fatigue performance of high strength steel built-up box T-joints. J. Constr. Steel. Res..

[15] Girão Coelho A.M., Bijlaard F.S.K. (2007). Experimental behavior of high strength steel end-plate connection. J. Constr. Steel. Res..

[16] Su J., Li Z., Wang J., Dhakal R.P. (2020). Numerical simulation and damage analysis of RC bridge piers reinforced with varying yield strength steel reinforcement. Soil. Dyn. Earthq. Eng..

[17] Yin H., Teo W., Shirai K. (2017). Experimental investigation on the behavior of reinforced concrete slabs strengthened with ultra-high performance concrete. Constr. Build. Mater..

[18] Zhang Y., Cai S., Zhu Y., Fan L., Shao X. (2020). Flexural responses of steel-UHPC composite beams under hogging moment. Eng. Struct..

[19] Qi J., Cheng Z., Wang J., Tang Y. (2020). Flexural behavior of steel-UHPFRC composite beams under negative moment. Structures.

[20] Shafieifar M., Farzad M., Azizinamini A. (2018). A comparison of existing analytical methods to predict the flexural capacity of Ultra High Performance Concrete (UHPC) beams. Constr. Build. Mater..

[21] Xu M., Liang X., Wang P., Wang Z. (2019). Theoretical investigation on normal section flexural capacity of UHPC beams. Eng. Mech..

[22] Xu M., Liang X., Yu J., Li L. (2019). Theoretical and experimental investigation on immediate stiffness of UHPC beams. Eng. Mech..

[23] Dogu M., Menkulasi F. (2020). A flexural design methodology for UHPC beams posttensioned with unbonded tendons. Eng. Struct..

[24] Zhang Y., Zhu Y., Yeseta M., Meng D., Shao X., Dang Q., Chen G. (2019). Flexural behaviors and capacity prediction on damaged reinforcement concrete (RC) bridge deck strengthened by ultra-high performance concrete (UHPC) layer. Constr. Build. Mater..

[25] Yan P., Chen B., Afgan S., Aminul Haque M., Wu M., Han J. (2021). Experimental research on ductility enhancement of ultra-high performance concrete incorporation with basalt fiber, polypropylene fiber and glass fiber. Constr. Build. Mater..

[26] Yang I., Park J., Bui T., Kim K., Joh C., Lee H. (2020). An experimental study on the ductility and flexural toughness of ultrahigh-performance concrete beams subjected to bending. Materials.

[27] Khaksefidi S., Ghalehnovi M., De Brito J. (2021). Bond behavior of high-strength steel rebars in normal (NSC) and ultra-high performance concrete (UHPC). Constr. Build. Mater..

[28] Tong L., Chen L., Wen M., Xu C. (2020). Static behavior of stud shear connectors in high-strength-steel–UHPC composite beams. Eng. Struct..

[29] Saleem M.A., Mirmiran A., Xia J., Mackie K. (2011). Ultra-High-Performance Concrete Bridge Deck Reinforced with High-Strength Steel. Aci. Struct. J..

[30] Xia J., Mackie K.R., Saleem M.A., Mirmiran A. (2011). Shear failure analysis on ultra-high performance concrete beams reinforced with high strength steel. Eng. Struct..

[31] Ghasemi S., Zohrevand P., Mirmiran A., Xiao Y., Mackie K. (2016). A super lightweight UHPC–HSS deck panel for movable bridges. Eng. Struct..

[32] Zhang J., Liu J., Li X., Cao W. (2021). Seismic behavior of steel fiber-reinforced high-strength concrete mid-rise shear walls with high-strength steel rebar. J. Build. Eng..

[33] Perceka W., Liao W. (2021). Experimental study of shear behavior of high strength steel fiber reinforced concrete columns. Eng. Struct..

[34] He S., Deng Z., Yao J. (2020). Seismic behavior of ultra-high performance concrete long columns reinforced with high-strength steel. J. Build. Eng..

[35] Sharifa A.M., Assi N., Al-Osta M. (2020). Use of UHPC slab for continuous composite steel-concrete girders. Steel. Compos. Struct..

[36] (2015). Reactive Powder Concrete.

[37] (2016). Concrete—Ultra-High Performance Fiber-Reinforced Concrete—Specifications, Performance, Production and Conformity.

[38] Wang W., Zhao G., Li G., Weng C. (2003). Experimental study on Flexural behavior of reinforced concrete beam reinforced with glass fiber Cloth. J. Dalian. Univ. Technol..

[39] Djamaluddin R. (2013). Flexural behaviour of external reinforced concrete beams. Procedia. Eng..

[40] Sun Q., Liu C. (2021). Experimental study and calculation method on the flexural resistance of reinforced concrete beam strengthened using high strain-hardening ultra high performance concrete. Struct. Concrete..

[41] Xu H. (2015). Study on the Mechanical Behavior of HRB500 Reinforced Prestressed Ultra-high performance Concrete Beam.

[42] Luo M., Lin P., Yang Z. (2020). Study of Mechanical Properties and Constitutive Relations of UHPC under Uniaxial Compressive Loading. Bridge Constr..

[43] Guo X., Kang J., Zhu J. (2017). Constitutive relationship of ultrahigh performance concrete under uni-axial compression. J. Southeast. Univ..

[44] Yang J., Fang Z. (2008). Research on stress-strain relation of ultra high performance concrete. Concrete.

[45] Hu A., Liang X., Yu J., Shi Q. (2018). Tensile characteristics of ultra-high-performance concrete. Mag. Concrete. Res..

[46] Zhang Z., Shao X., Li W., Zhu P., Chen H. (2015). Axial Tensile Behavior Test of Ultra High Performance Concrete. China J. Highw. Tranp..

[47] Meng W., Khayat K.H. (2018). Effect of hybrid fibers on fresh properties, mechanical properties, and autogenous shrinkage of cost-effective UHPC. J. Mater. Civ. Eng.

[48] Feng P., Qiang H., Ye L. (2017). Discussion and definition on yield points of materials, Members and Structures. Eng. Mech..

